# QTL architecture of reproductive fitness characters in *Brassica rapa*

**DOI:** 10.1186/1471-2229-14-66

**Published:** 2014-03-18

**Authors:** Jennifer M Dechaine, Marcus T Brock, Cynthia Weinig

**Affiliations:** 1 Department of Biological Sciences, Central Washington University, Ellensburg, WA 98926, USA; 2 Department of Botany, University of Wyoming, Laramie, WY 82071, USA

**Keywords:** Fitness components, Life-history traits, Phenotypic plasticity, Transgenerational effects, Yield, *Brassica rapa*

## Abstract

**Background:**

Reproductive output is critical to both agronomists seeking to increase seed yield and to evolutionary biologists interested in understanding natural selection. We examine the genetic architecture of diverse reproductive fitness traits in recombinant inbred lines (RILs) developed from a crop (seed oil) × wild-like (rapid cycling) genotype of *Brassica rapa* in field and greenhouse environments.

**Results:**

Several fitness traits showed strong correlations and QTL-colocalization across environments (days to bolting, fruit length and seed color). Total fruit number was uncorrelated across environments and most QTL affecting this trait were correspondingly environment-specific. Most fitness components were positively correlated, consistent with life-history theory that genotypic variation in resource acquisition masks tradeoffs. Finally, we detected evidence of transgenerational pleiotropy, that is, maternal days to bolting was negatively correlated with days to offspring germination. A QTL for this transgenerational correlation was mapped to a genomic region harboring one copy of *FLOWERING LOCUS C*, a genetic locus known to affect both days to flowering as well as germination phenotypes.

**Conclusions:**

This study characterizes the genetic structure of important fitness/yield traits within and between generations in *B. rapa*. Several identified QTL are suitable candidates for fine-mapping for the improvement of yield in crop *Brassicas.* Specifically, *brFLC1*, warrants further investigation as a potential regulator of phenology between generations.

## Background

Female reproductive fitness is an important measure of overall crop yield for many cultivated species, such as grain crops and oilseeds. In an evolutionary context, reproductive fitness is commonly used to estimate selection, as in studies investigating crop allele persistence in wild populations [1-3]. Fitness and yield are determined by complex interactions among traits expressed throughout plant development, such as flowering time [4,5], fruit shape [6], seed size [7,8] and seed number [9]. Recent quantitative trait loci (QTL) mapping studies in several agricultural systems have suggested that QTL affecting fitness traits are often clustered in the genome, suggesting tight physical linkage or pleiotropic effects on several components of fitness [9-14], but the ubiquity of this result across experimental populations and environments is not well understood.

Elucidating the genetic architecture underlying fitness traits is critical to predicting their response to selection. For example, although it may be desirable to increase both seed size and seed number in oilseed cultivars, such as *Brassica rapa*, selection for these traits may be constrained if they are controlled by antagonistic pleiotropy of a single locus or by loci in repulsion phase and in such close physical proximity that recombination is limited. A QTL mapping study in *Arabidopsis thaliana* suggested that antagonistic pleiotropy of a gene region controlling both seed mass and ovule number may lead to the trade-offs between these traits that have been observed in numerous cultivated and natural species [7]. If this region represents structured pleiotropy, in that allelic substitutions at one locus affect both traits, they will be resistant to change even under strong selection [15,16]. In this situation, it may be prudent to search for natural populations in which these associations are weaker or focus selection efforts on loci that are not pleiotropically regulated.

If covariances among desirable traits are primarily due to extensive linkage disequilibrium, fine-mapping through marker-assisted selection could help to disrupt these associations [17]. QTL regions often encompass regions too large to conclusively discriminate between pleiotropy and linkage, but they provide a starting point for fine-mapping and marker-assisted selection of specific loci [18,19]. Recent studies have advanced from QTL screens to map-based cloning and association mapping to successfully identify candidate genes for important fitness traits in seed oil crops, such as *Brassica* species [6,20-23].

It is also of interest to understand how QTL expression for fitness characters may vary across simulated versus field/natural settings, as plant breeders attempt to extrapolate to the field from controlled experiments. Significant QTL × environment interactions have been detected for fitness traits in *A. thaliana* plants grown in the field versus greenhouse or growth chamber environments, suggesting significant differences in QTL expression between controlled and natural settings [24-26]. On the other hand, strong genetic correlations and QTL colocalization between the greenhouse and field have been observed for traits with high heritabilities [26]. Extrapolating QTL data from controlled to field environments may thus be appropriate for some traits.

Seed germination is an important but frequently neglected component of plant fitness. Establishment is a more important determinant of the composition of natural plant populations than is seed availability in many species [27,28]. Germination timing influences fitness by altering the expression of flowering time and other important life-history characters in the annual plant species, *Arabidopsis thaliana* and *Campanulastrum americanum*[29-31]. In *A. thaliana,* seed dormancy QTL colocalize with QTL affecting total lifetime fitness, and epistatic loci affect both traits under field conditions [32]. Clearly, germination phenology influences plant fitness within a generation, but it is equally sensitive to transgenerational influences, which are less well understood, especially in regard to their genetic basis.

Germination phenology is highly sensitive to the maternal genotype, phenotype, and environment [30,33-41]. Maternal flowering day and subsequent timing of seed dispersal can alter germination season in the next generation [34]. Seed size may also affect germination. Studies have found a positive association between seed size and germination percentage or timing in numerous plant species [42], although faster emergence times in smaller seeds have also been demonstrated [43-45]. Despite extensive ecological correlations between seed characters and germination, few studies have mapped QTL for these traits within the same study, and those that have found no QTL affecting both seed size and germination percentage or rate [25,46]. In addition, we are unaware of any study mapping QTL for maternal flowering and progeny germination phenology.

In this study, we examine the quantitative-genetic structure and QTL architecture of bolting time and reproductive fitness characters in recombinant inbred lines of *Brassica rapa* developed from a cross between a seed oil cultivar and wild-like genotype. QTL mapping was conducted in similar greenhouse and field experiments in order to assess how accurately QTL studies in simulated environments can be extrapolated to the field. In the greenhouse environment, we also examine the relationship between germination phenology and the maternal characters. *Brassica rapa* is an internationally cultivated seed oil and vegetable crop and a parent of the hybrid-origin mustard crops, *B. napus* (canola) and *B. juncea* (Indian mustard) [47]. Numerous genetic resources have been developed for this species [22,23,48,49], and the first reference sequence for *B. rapa* was released in 2011 [50]. Naturalized populations of *B. rapa* are distributed throughout the United States and often occur in disturbed habitats or the margins of agricultural fields [51,52]. Wild *B. rapa* are highly phenotypically variable, ranging from smaller, rapidly-developing spring annuals to larger biennials [52]. Although QTL affecting fruit and seed traits have been identified in a few *B. rapa* cultivars [53], no study mapping reproductive fitness QTL in a cross between a seed oil cultivar and a wild-like population has been reported.

## Methods

### Study system

The recombinant inbred lines (RILs) used in this study have been previously described in detail [48,54,55]. Briefly, the RILs were derived from a cross between two inbred *B. rapa* lines, the seed oil cultivar, yellow sarson (R500), and a rapid-cycling genotype (IMB211). Yellow sarson is a highly inbred, annual *B. rapa* cultivated in India for over 3,000 years [56]. IMB211 is derived from the Wisconsin Fast Plant™ (WFP) population produced by selection for short generation time [57]. The parental genotypes differ greatly for several reproductive fitness traits. R500 produces the large, heavy, yellow seeds typical of *B. rapa* cultivars, and IMB211 produces smaller, lighter, brown seeds more similar to naturalized *B. rapa* populations. IMB211 is phenotypically similar to rapid-developing *B. rapa* in the United States, and its average flowering day and height are within the range of wild-collected *B. rapa*[52]. The RILs were developed from self-fertilization of a F1 individual resulting from the R500 × IMB211 cross, and the resulting F2s were propagated by single-seed descent to the S6 generation [48].

### Experimental environments

Seeds of 147 RILs and the parental lines were planted in an agricultural field at the University of Minnesota, Saint Paul, MN, April 29–31, 2004. In each of three subplots, four plants of each RIL were planted at 20 cm spacing from each other and the next RIL, resulting in three sets of four adjacent replicates within each RIL. This experiment was part of a larger one examining multiple competitive environments, and further details of the design have been previously described [55]. Bolting date was scored as the date when buds first differentiated from the apical meristem. All flowers were naturally open-pollinated in the field. Plants began to naturally senesce (leaves and stem began to turn brown and dry) in the first week of August and were harvested once 50% of the rosette leaves had senesced on all individuals. At harvest, fruits (siliques) were counted on each plant, and four mature (fully developed) fruits were collected at random from 3–5 replicates of a RIL. Seeds were counted in each fruit, and the length of the longest fruit was measured. Seeds were then pooled within a plant, and seeds-per-fruit, seed mass, seed area, and seed color were determined. Seeds-per-fruit is the mean number of seeds in the 2 longest collected fruit. Seed mass was calculated as the average mass of 20 randomly chosen seeds from the collected fruit. Ten seeds from each plant were then photographed, and their area and color were determined using ImagePro software, which quantifies pixels in digital photographs of the sample (ImagePro Plus V4.5 2001). The trait, seed color, was obtained as the score of the first principal component identified in principal components analysis, using varimax rotation, on pixel densities of three colors: red, green, and blue (PROC FACTOR; SAS, 2001). The first principal component, which was weighted most heavily in red and green, explained greater than 67% of the variation. A lower value for seed color indicates darker, more wild-like seeds.

Fitness traits were also recorded in a greenhouse experiment. Due to space limitations, the greenhouse experiment was conducted over two temporally separated trials, spaced a month apart. In the first trial, on March 14^th^, 2007, we planted 8 replicates of each of the parental lines and 137 of the 160 *B. rapa* RILs into 3 in^3^ pots, filled with Metromix 200 soil, in greenhouses at the University of Minnesota, Saint Paul, MN. Bolting date was recorded for each individual using the same method as in the field, and these plants were then harvested.

Seeds were planted for the second experimental trial on April 17th, 2007 using the same RILs and in the manner described above. Six replicates per line were planted. At 5 days after bolting, plants began flowering, and the number of open flowers was recorded daily. The majority of flowers were allowed to self-pollinate naturally, except that flowers at the 6^th^ and 7^th^ nodes were self-fertilized by hand in order to more closely simulate the effect of pollinators in the field. In early June, the plants began to senesce. At senescence, fruits were counted on each plant, and fruits 6 and 7 (or those fruits arising from hand-pollination) were collected. Fruit length was measured on the longest of the fruits, and the seeds in each collected fruit were counted. Seeds were pooled within a plant, and seed traits were measured as in the field.

Germination characteristics of the seeds collected in the greenhouse experiment were also examined. Seeds collected from the 2 parental lines and RILs with at least 3 replicate plants that set seed were pooled and stored in darkness at room temperature. While excluding RILs with fewer than 3 replicate plants artificially eliminates lines with low viability, this sampling approach helps reduce microenvironmental bias. To further limit microenvironmental maternal effects, the same number of seeds were pooled from each replicate within a RIL combination if possible. Six replicates of 5–10 seeds per RIL were germinated in 3 Conviron E7/2 growth chambers (Controlled Environments Ltd., Winnipeg, Canada) in 2 temporal blocks from February 1–12, 2007 and from March 20–30, 2007. One replicate of each RIL was located in each growth chamber and temporal block, and replicates were randomized within a growth chamber. All seeds were planted onto 0.5% agar in 4.5 cm diameter plastic Petri dishes under a green safe light and then allowed to imbibe water for 24 h in darkness before being moved into the growth chambers. Each growth chamber was fitted with 8 white florescent lights (Silvania Octron, F038/871, 32 W), 2 infrared lights (Industrial Infrared, F32T8/IR-750), and 4 incandescent lights (Sylvania Double Life, Soft White, 120 V/75 W) that generated a R:FR of 1.1 and PAR of 130 – 160 μmol m^-2^ s^-1^. Germination trials used 12 L:12D lighting and temperatures of 24D:20 N °C. Germination was checked daily for 10 days and scored when the cotyledons appeared green and open to ~90°. Germination percent and day were calculated by dividing the number of seeds germinated by the seed total and by averaging the germination days for all seeds in a replicate Petri dish, respectively.

### Quantitative genetic analysis

For all traits, a restricted maximum likelihood (REML) approach (PROC MIXED, SAS 2001) was used to partition variation attributable to RIL (V_L_), spatial blocking (V_B_), and residual error (V_R_) within each environment. The blocking term was subplot for all traits in the field and greenhouse, and temporal block and chamber for the germination traits measured in the growth chamber. Seed color and fruit length met the assumption of normality in both the field and the greenhouse environments, but all remaining traits were transformed using a Box-Cox procedure [58]. All transformations greatly improved normality and homoscedasticity, and results from the transformed data are reported.

Broad-sense heritabilities were calculated as V_L_/V_P_, where, again,V_L_ equals the among-RIL variance component and V_P_ equals the total phenotypic variance for a trait. Least-square means, 95% confidence limits, and best linear unbiased predictor (BLUP) deviations were generated for each RIL using the transformed versions of the traits. BLUP deviations and least-square means were summed, and the resulting values were back-transformed for use in the QTL analysis. The parental genotypes, plants that died before bolting, and RILs with fewer than 3 replicates for a trait were removed from the analyses. Final sample sizes ranged from 730 to 1160 (out of a possible 3600) in the field and 910 to 1400 (out of a possible 2466) in the greenhouse. BLUPs were also used to estimate genetic correlations (r_G_) within each environment (PROC CORR, SAS 2001). Differences in bivariate matrix structure across environments was tested using Flury’s hierarchical common principal components (CPC) analysis, using the "jump-up approach" and Fisher’s Z-test [59-61]. Cross-environment correlations (r_GE_) for each trait were calculated as cov_1,2_/√ (V_1,1_ × V_1,2_); cov_12_ is the covariance of a trait across two environments and V_1,1_ and V_1,2_ are the among-RIL variances within each environment [62,63].

*QTL-analysis.* The R500 × IMB211 RILs were genotyped at 227 RFLP and SSR markers across 10 linkage groups, representing the 10 chromosomes in *B. rapa*. Marker order for the linkage map was estimated from recombination frequencies observed in the entire population of 160 RILs, i.e., from the most complete dataset, in earlier experiments [48]. Centimorgan distances were then re-estimated in the RILs included in the field and greenhouse experiments presented here (those that survived to bolting) to account for slight differences that might exist due to unsampled recombination events. The map distances for each marker locus were calculated from the estimated recombination frequencies using the Kosambi mapping function in RQTL (R Development Core Team 2010). Twelve markers were uninformative, leaving 215 markers at unique positions in the final re-estimated linkage map.

QTL were mapped using the composite interval mapping (CIM) procedure in QTL Cartographer [64]. To control for effects of variation segregating elsewhere in the genome, we identified co-factors using forward-backward regression, and a 5-cM window; a maximum of 5 co-factors was selected for inclusion in the mapping model. We scanned for QTL at 2-cM intervals across the *B. rapa* genome. The significance threshold of the likelihood-ratio test statistic (LR) was determined for each trait in each treatment independently by randomly permuting the BLUPs 1000 times. QTL with LRs significant at α = 0.05 are reported [65] with 2-LOD support limits [66]. Additive effects and the genetic variance explained by a QTL were calculated in QTL Cartographer, and confirmed using a GLM model with all QTL detected in the genome-wide screen as main effects [67]. For a given locus, a positive additive effect indicates that the IMB211 parent allele conferred a higher value for the trait. Analysis of variance (ANOVA) was used to test for QTL × environment interactions across the field and greenhouse environments; all significant main-effect QTL for the trait, environment, and QTL × environment effects on each genotypic trait mean were included in the model (PROC GLM, SAS 2001).

## Results

### Descriptive statistics

**Figure 1:**
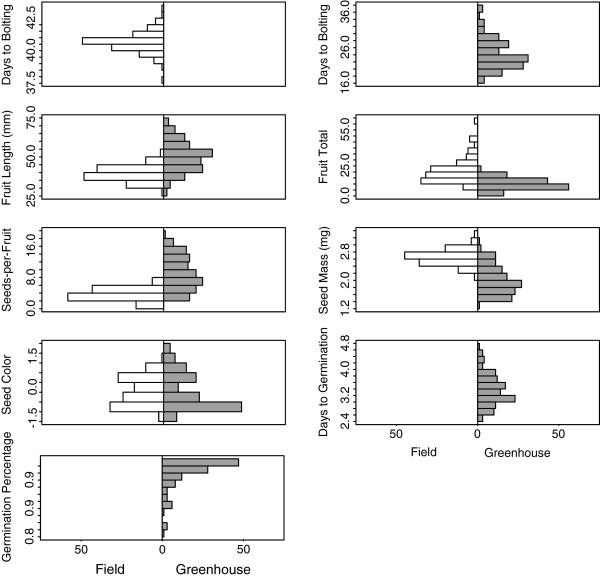
**Frequency histograms of best-linear unbiased predictors for reproductive fitness traits in the *Brassica rapa *R500 × IMB211 recombinant inbred line population field and greenhouse environments.** Seed color is the first principal component; a lessor value indicates darker, more wild-like seeds. Germination traits were not measured in the field.

**Figure 2:**
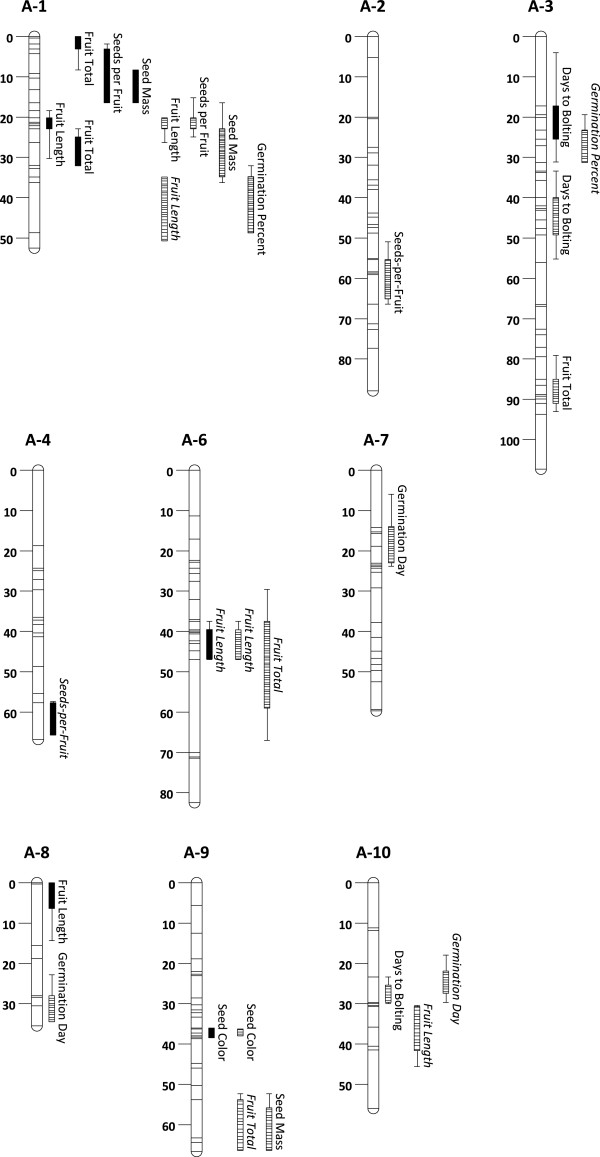
**QTL in the field (filled) and greenhouse (dashed) environments with 1-LOD (bars) and 2-LOD (tails) support limits.** Positive additive effects of the IMB211 (wild-like) parent allele are indicated with italics.

RILs varied significantly (*P* < 0.001) in phenotypic expression of all traits in both environments, indicating underlying genetic variation (Additional file 1: Table S1; Figure 1). Broad-sense heritabilities (*H*^*2*^) were smaller in the field (range, 0.08-0.79) than the greenhouse (range, 0.26-0.86) for all traits except fruit total (Table 1). We detected 7 QTL affecting 6 traits in the field and 12 QTL affecting 8 traits in the greenhouse (Table 2A and B; Figure 2). For any trait, a single QTL explained 5–56% (greenhouse) or 5–59% (field) of the variance (Table 2). Alleles derived from IMB211 had both positive and negative effects on the majority of traits with > 1 QTL in one or both environments, with the exception that the IMB211 allele always decreased the trait value for days to bolting, seed mass, and seed color.

**Table 1:** Quantitative genetic partitioning of variance components and trait means in the field (F) and greenhouse (GH) environments

	**V**_**L**_		**V**_**R**_		***H***^***2 ***^**(V**_**L**_**/V**_**P**_**)**		**Means (CL)**	
**Trait**	**F**	**GH**	**F**	**GH**	**F**	**GH**	**F**	**GH**
Days to bolting	0.79	0.00	5.07	0.00	0.13	0.71	40.69	23.65
							(41.12-40.26)	(21.95-25.63)
Fruit length	36.21	106.94	69.00	71.44	0.33	0.60	39.07	50.38
							(37.3-40.84)	(48.30-52.47)
Fruit total	0.48	0.64	1.05	1.36	0.30	0.30	19.12	9.44
							(15.22-24.01)	(7.78-11.26)
Seeds-per-fruit	0.03	0.57	0.31	0.77	0.08	0.42	3.41	8.52
							(2.31-4.85)	(7.49-9.61)
Seed mass	0.01	0.02	0.03	0.02	0.23	0.49	2.64	1.99
							(2.48-2.81)	(1.86-2.14)
Seed color	0.59	0.86	0.15	0.14	0.79	0.86	0.04	0.06
							(-0.11-0.19)	(-0.12-0.23)
Days to germination	-	0.00	-	0.00	-	0.26	-	3.33
								(1.50-12.92)
Germination percentage	-	0.02	-	0.04	-	0.30	-	0.98
								(0.75-1.11)

Restricted maximum likelihood estimates of the among-RIL variance component (V_L_), the residual variance component (V_R_), and the broad sense heritability (*H*^*2*^), calculated as V_L_/(V_P_); V_P_ is the total phenotypic variance component. Least-squared means (Means) and 95% confidence limits (CL). Length and height traits are in mm. Seed area and seed mass are mm^2^ and mg, respectively. Fruit total and seed total are per plant. Seed color is a single factor principal component of red, green, and blue colors; a negative number indicates darker, more brown seeds. Germination percentage indicates the number of seeds that germinated within 10 days from planting. Germination traits were not assessed in the field.

**Table 2:** QTL mapping in the field (A) and greenhouse (B)

**QTL**	**Trait**	**Marker**	**2-LOD**	**α/SD**	**PVE**
A.					
FQTL1-1	Fruit total	**pW249dX**	**0.00-8.28**	**-0.28**	**7.40**
	Seeds-per-fruit	fito133a	1.92-16.52	-0.31	9.50
	Seed mass	pX106aH	8.28-16.52	-0.53	22.20
FQTL1-2	Fruit length	fito083	18.37-30.34	-0.41	15.70
	Fruit total	**pX136bE**	**22.87-32.06**	**-0.33**	**10.60**
FQTL3-1	Days to bolting	pX144bE	4.01-31.12	-0.33	10.72
FQTL4-3	Seeds-per-fruit	pW178bE	57.41-65.67	0.31	8.70
FQTL6-1	Fruit length	pX136dE	37.46-47.02	0.27	7.40
FQTL8-1	Fruit length	BRMS006	0.01-14.35	-0.28	7.60
FQTL9-1	Seed color	fito555	38.36-40.67	-0.78	58.70
B.					
GHQTL1-1	Seeds-per-fruit	fito083	15.23-24.87	-0.40	8.60
	Seed mass	pX136bE	16.52-36.27	-0.36	15.40
	Fruit length	pW108aE	20.20-26.34	-0.47	21.40
GHQTL1-2	Germination percentage	pX122aH	32.06-48.72	-0.22	8.50
	Fruit length	pX122aH	34.87-50.72	0.27	6.50
GHQTL2-1	Seeds-per-fruit	**fito473**	**50.86-66.40**	**-0.30**	**12.20**
GHQTL3-1	Germination percentage	pW152cH	19.39-31.30	0.43	10.38
	Days to bolting	fito071d	33.44-55.17	-0.24	5.26
GHQTL3-3	Fruit total	pW177aH	79.12-93.03	-0.36	12.60
GHQTL6-1	Fruit total	fito227	29.60-67.02	0.30	8.50
	Fruit length	pX136dE	37.46-47.02	0.24	5.70
GHQTL7-1	Days to germination	pW108aH	6.01-23.81	-0.36	8.50
GHQTL8-1	Days to germination	pW245bX	22.79-34.50	-0.27	10.40
GHQTL9-1	Seed color	fito367b	36.34-38.01	-0.78	56.10
GHQTL9-2	Fruit total	**fito151a**	**52.29-66.41**	**0.34**	**11.70**
	Seed mass	**pW246cX**	**52.29-66.41**	**-0.31**	**7.10**
GHQTL10-1	Days to germination	pW155cX	17.91-29.69	0.46	9.80
	Days to bolting	**pW155cX**	**23.43-30.04**	**-0.53**	**26.39**
GHQTL10-2	Fruit length	**pW129dH**	**30.38-45.55**	**0.36**	**11.70**

QTL are significant at α = 0.05. QTL names indicate the chromosome number - QTL number.

Columns 3 and 4 show the left-flanking marker for each QTL and the range of the 2-LOD support limits in cM. Significant QTL x environment interactions between environments are in boldface. Columns 5 and 6 indicate the standardized additive effect (α/standard deviation) of the IMB211 allele and the percent variance explained (PVE) by the QTL.

### Genetic architecture between environments

Single trait genotypic correlations between environments were positive and significant for days to bolting, fruit length, seed mass, and seed color (Table 3). Consistent with the correlations, the majority of QTL for these traits did not exhibit significant QTL × environment interactions (Table 2). Seed color had the highest heritability and cross-environment correlation, and we detected only one large-effect QTL for this trait that was expressed in both environments. These results suggest that the QTL architecture for the majority of fitness traits examined in this study was at least partially conserved between the greenhouse and field.

**Table 3:** Broad-sense genotypic correlations among traits in the field (above diagonal) and greenhouse (below diagonal) environments

	**Days to bolting**	**Fruit length**	**Fruit total**	**Seeds-per-fruit**	**Seed mass**	**Seed color**	**Days to germ**
Days to bolting	0.56****	-0.03	0.14	0.22*	-0.11	**-0.14**	-
Fruit length	-0.17*	0.61****	0.28***	0.27***	0.41****	0.00	-
Fruit total	0.08	0.16^	-0.04	0.20*	0.33**	**0.13**	-
Seeds-per-fruit	**-0.04**	0.59****	0.32****	0.12	0.35****	-0.32**	-
Seed mass	-0.07	0.20*	**-0.06**	0.06	0.25*	**-0.09**	-
Seed color	**0.12**	-0.05	**-0.12**	-0.21*	0.24**	0.80****	-
Days to germination	-0.31**	0.17^	-0.16	0.00	0.21*	-0.05	-
Germ percentage	0.10	0.08	0.00	0.25**	0.26**	0.09	-.16^

Cross-environmental correlations for each trait are on the diagonal. Pearson correlation coefficients are shown. Asterisks indicate significance: ^<0.07; *P<0.05; **P <0.01; ***P <0.001; ****P <0.0001. Boldface indicates Z-test significance differences at *P* <0.05 across environments for bivariate correlations.

By contrast, genotypic correlations were non-significant for fruit total between environments (Table 3). Three of the five QTL affecting fruit total, including all QTL detected in the field for this trait, were environment-specific, in that the QTL × environment effects were significant (Table 2). QTL × environment effects were also significant for one QTL affecting each of days to bolting, fruit length, seeds-per-fruit, and seed mass, indicating that at least some of the genetic loci affecting each reproductive character, except seed color, were environment-specific or that functional differences between alleles at these QTL were manifested only in one environment.

### Relationship among components of fitness

A number of bivariate correlations were conserved between environments. RILs that produced more fruit also produced more seeds-per-fruit, and longer fruits generally had heavier seeds and more seeds (although the fruit length/seeds-per-fruit correlation was significantly stronger in the greenhouse) (Table 3). Plants that produced more seeds-per-fruit also had darker seeds (negative value for seed color) in both environments.

Environmental differences in bivariate correlations were also observed. Plants that produced more fruit and more seeds-per-fruit also had heavier seeds in the field, but these correlations were not significant in the greenhouse (although the difference between environments was not large enough to be statistically significant for the seeds-per-fruit/seed mass correlation). These results suggest that genotypic differences in resource acquisition existed among plants grown in the field, that is, genotypes with high acquisition had higher output for all reproductive components than those with lower acquisition. In the greenhouse, plants did not generally produce both more seeds and larger seeds, suggesting that the genotypic differences in acquisition were smaller in this environment. The relationship between seed mass and color also differed between environments. Although the bivariate correlation between seed mass and color was positive in the greenhouse, the scatter plot showed a sharp increase in seed mass in the darkest colored seeds (Additional file 2: Figure S1), indicating that the lightest and darkest seeds had the largest mass. This relationship was not conserved in the field. Interestingly, significant Z-test differences indicated that the relationship between seed color and seed mass or fruit total reversed between environments, although the relationship between these traits was too weak in each environment for the majority of bivariate correlations to be significant.

The field-specific positive relationship between fruit total, seeds-per-fruit, and seed mass was consistent with FQTL1-1, which decreased the value for all three traits in the field and had a significant QTL × environment interaction effect for fruit total (Table 2). Moreover, GHQTL9-2 had opposing effects on fruit total versus seed mass, and the QTL × environment effects were significant for these traits (Table 2; Figure 2). These results indicate that GHQTL9-2 conferred environment-specific differences in resource allocation to reproductive fitness traits.

### Transgenerational fitness

In the greenhouse environment, we examined the relationships between reproductive characters of the maternal parent and germination patterns in the progeny. Interestingly, later bolting maternal plants produced faster germinating seeds, suggesting a phenological relationship across generations (Table 3). This relationship was consistent with GHQTL10-1, which increased time to germination but decreased time to bolting (Table 2; Figure 2), providing evidence of transgenerational antagonistic pleiotropy at this locus. We also observed a positive relationship between both seed mass and seeds-per-fruit and germination percentage, suggesting that more vigorous maternal genotypes partition more resources to offspring and produce many large seeds that also germinate to a higher percentage.

## Discussion

In the current study, we examined the genetic architecture of reproductive fitness components, a topic of interest to both agronomists seeking to increase yield and evolutionary biologists interested in predicting response to selection in quantitative traits. QTL that are consistently expressed among environments are favorable targets for marker-assisted selection in the development of widely distributed agricultural varieties [9]. We detected QTL affecting seeds-per-fruit, seed mass, and fruit length on A1 in both the greenhouse and field environments. These results are consistent with a fruit length QTL previously mapped near the center of A1 in *B. rapa*[53,68], as well as QTL affecting seed mass [6] and multiple reproductive fitness traits on A1 in *B. napus*[9,69]. Shi et al. (2009) detected several linked consensus QTL each affecting multiple seed or fruit traits on A1 in *B. napus*, indicating significant pleiotropy and physical linkage among fitness loci on this chromosome. Given the large influence of sections of A1 on reproductive fitness and yield, this chromosome should be a primary target for fine-mapping and candidate gene screening using association analyses in *Brassica* species*.*

Aside from the number and size of seeds, seed oil is a common target of selection among *B. rapa* varieties, and selection for seed oil phenotypes leads to a differentiation in seed color between cultivated and wild genotypes [70,71]. In the current study, seed color was highly conserved across environments. Heritability and the cross-environment correlation for this trait were both over 0.8, and we detected one large-effect QTL affecting only this trait on A9 in both environments. This supports previous studies mapping seed color to one or a few genes on A9 in *B. rapa*[23,49]. The candidate gene *BrTT8* has been identified in this region and shown to control the accumulation of proanthocyanidins in the seed coat [71,72]. These results suggest that QTL mapping data from greenhouse studies can be extrapolated to field work for loci with a large additive genetic component.

Despite some shared genetic control, at least one QTL for all traits other than seed color was detected in only one environment, suggesting environment-specific effects on fitness traits. Fruit total was largely environment-specific; the genotypic correlation across environments was non-significant for fruit total and seeds-per-fruit, and we detected significant QTL × environment effects for 3 of the 5 QTL affecting fruit total. These results indicate that the genetic architecture of overall reproductive output (total fruit production) is more environmentally sensitive than other seed and fruit traits. A recent meta-analysis of QTL in *B. napus* that found that 63% of QTL affecting total seed yield were microenvironment-specific versus 47% of QTL for all fitness traits measured [9]. Moreover, 85% of seed yield QTL colocalized with QTL for at least one other life history or reproductive fitness trait [9]. In the current study, all but 1 fruit total QTL affected multiple traits, indicating that different aspects of reproductive output are determined by multiple closely linked or pleiotropic loci.

Fine-mapping studies have found clustered, pleiotropic QTL affecting various life history and fitness characters in rice [5], wheat [73], pea [74], and *B. napus*[9]. Selection for any one of these QTL is likely to have far reaching consequences on plant fitness. If, for example, QTL clusters correspond to genes regulating coordinated steps in seed development, then selection may have desirable outcomes on several traits, such as an increase in seed size and protein content [74]. Alternatively, antagonistic associations among QTL, as has been found for oil traits in *B. juncea*[75], may result in negative, nontarget consequences on desirable traits. In our study, a parental allele generally had the same direction of effect on all traits at a QTL, thus supporting coordinated gene regulation of reproductive fitness traits. The only exception was GHQTL9-2, which had opposing effects on fruit total and seed mass in the greenhouse environment, leading to conditional neutrality in the relationship between these traits that differed between the greenhouse and field [76]. Moreover, the opposing effects of GHQTL9-2 on fruit total and seed mass suggests that antagonistic pleiotropy may control allocation of resources to seed size versus seed production in *B. rapa,* similar to what has been described in *A. thaliana*[7].

In natural systems, seed germination and establishment are no less important indicators of reproductive fitness than seed traits. Our study provides QTL support for transgenerational co-regulation of life history phenology. Later bolting maternal plants produced seeds that germinated earlier, consistent with opposing effects of SQTL10-1 on these traits. Our results support previous work demonstrating an adaptive association between maternal flowering time and germination season in *Campanulastrum americanum*[30,34]. Early flowering *C. americanum* plants produce seeds that disperse earlier and thus germinate earlier because of environmental cues, and strong negative genetic correlations between maternal flowering day and offspring germination timing have been observed in this species [35]. Our results provide QTL support for negative across-generation co-regulation of flowering and germination timing in *B. rapa*, similar to what has been observed in *C. americanum.*

The observed negative relationship between days to bolting and germination timing in our study was consistent with opposing effects of SQTL10-1 on these traits, suggesting that in this region there is an important regulatory locus or cluster of loci influencing phenology between generations. SQTL10-1 was flanked by *BrFLC1*, which is collinear to *FLC* in *A. thaliana*[77]. *A. thaliana FLC* has been shown to act through the flowering time pathway to maternally-regulate germination [78] and is subject to transgenerational epigenetic regulation [78-80]. In *B. rapa*, a study examining flowering time in a backcross population between biennial and annual *B. rapa* suggested that most of the variation in flowering time between these life histories was due to *FLC* loci, including *FLC1*[77]. Here, flowering time was variable, and it is unknown if SQTL10-1 would have affected germination time if flowering had been synchronous among RILs. *FLC1* is a likely candidate gene for transgenerational regulation of evolutionarily important life history phenology in multiple plant species and warrants further investigation of its direct and indirect (through flowering time) effects on germination under natural conditions.

## Conclusions

This is the first study to have characterized the correlation structure and QTL architecture of important fitness/yield traits in a crop × wild-like population of *B. rapa.* Days to bolting, fruit length, and seed color, were highly correlated and displayed QTL-colocalization across environments. Genetic characterization of these traits in controlled settings may readily translate to improvement in field environments. Total fruit number was uncorrelated across environments and most QTL affecting this trait were correspondingly environment-specific, suggesting that the genetic architecture of overall reproductive output is more environmentally sensitive than other measured fitness traits. Finally, *BrFLC1* was identified as a potential candidate gene affecting transgenerational regulation of flowering time and seed germination.

## Competing interests

The authors declare that they have no competing interests.

## Authors’ contributions

JMD, MTB, and CW designed research; JMD and MTB collected and analyzed data; JMD and CW wrote and prepared the manuscript. All authors read and approved the final manuscript.

## Supplementary Material

Additional file 1: Table S1 Tests of significance in field versus the greenhouse environments. Asterisks indicate P <0.001. Click here for file

Additional file 2: Figure S1 Scatterplot showing best-linear unbiased predictor values (BLUPs) for seed mass and seed color in the field (A) and greenhouse (B). A linear trendline (green) and lowess lines (red) are shown for each figure. Click here for file
