# Supplementary material for: QTL architecture of reproductive fitness characters in Brassica rapa
Source: BMC Plant Biol. 2014 Mar 18;14:66. doi: 10.1186/1471-2229-14-66 (PMC4004417; doi:10.1186/1471-2229-14-66)
Supplement: Additional file 2: Figure S1 — Scatterplot showing best-linear unbiased predictor values (BLUPs) for seed mass and seed color in the field (A) and greenhouse (B). A linear trendline (green) and lowess lines (red) are shown for each figure. [file 1471-2229-14-66-S2.docx]

Figure S1: Scatterplot showing best-linear unbiased predictor values (BLUPs) for seed mass and seed color in the field (A) and greenhouse (B). A linear trendline (green) and lowess lines (red) are shown for each figure.

Figure S1A.

Figure S1B.
